# Supplementary material for: Assessment of Maturity of Plum Samples Using Fourier Transform Near-Infrared Technique Combined with Chemometric Methods
Source: Foods. 2023 Aug 15;12(16):3059. doi: 10.3390/foods12163059 (PMC10453540; doi:10.3390/foods12163059)
Supplement: Supplementary file 1 [file foods-12-03059-s001.zip › foods-2541123-supplementary.pdf]

S1 Examination of reference data - Tukey (HSD) and Dunett (two sided) test/ Analysis of the differences between the categories with a confidence interval of 95%

Dry matter – DM

Summary of all pairwise comparisons for DM (Tukey (HSD)):

Category	LS means(DM)	Groups	
Immature	21,702	A	
Mature	18,712		B

Summary (LS means) - DM

	DM
Immature	21,702 [a]
Mature	18,712 [b]
Pr > F(Model)	< 0,0001
Significant	Yes

Titratable acidity TA

Summary of all pairwise comparisons for TA (Tukey (HSD)):

Category	LS means(TA)	Groups	
Immature	1,520	A	
Mature	0,891		B

Summary (LS means) - TA

	TA
Immature	1,520[a]
Mature	0,891[b]
Pr > F(Model)	< 0,0001
Significant	Yes

Total soluble solid – TSS

Summary of all pairwise comparisons for TSS (Tukey (HSD)):

Category	LS means(TSS)	Groups	
Mature	14,074	A	
Immature	8,742		B

Summary (LS means) - TSS

	TSS
1	14,074[a]
2	8,742[b]
Pr > F(Model)	< 0,0001
Significant	Yes

Maturity index MI=TSS/TA

Category	LS means(MI)	Groups	
Mature	16,383	A	
Immature	5,713		B

Summary (LS means) - MI

	MI
Mature	16,383 [a]
Immature	5,713 [b]
Pr > F(Model)	< 0,0001
Significant	Yes
